# Effect of Lysine to Arginine Mutagenesis in the V3 Loop of HIV-1 gp120 on Viral Entry Efficiency and Neutralization

**DOI:** 10.1371/journal.pone.0119879

**Published:** 2015-03-18

**Authors:** Birco Schwalbe, Michael Schreiber

**Affiliations:** Department Virology, Bernhard Nocht Institute for Tropical Medicine, Hamburg, Germany; Institute of Infection and Global Health, UNITED KINGDOM

## Abstract

HIV-1 infection is characterized by an ongoing replication leading to T-lymphocyte decline which is paralleled by the switch from CCR5 to CXCR4 coreceptor usage. To predict coreceptor usage, several computer algorithms using gp120 V3 loop sequence data have been developed. In these algorithms an occupation of the V3 positions 11 and 25, by one of the amino acids lysine (K) or arginine (R), is an indicator for CXCR4 usage. Amino acids R and K dominate at these two positions, but can also be identified at positions 9 and 10. Generally, CXCR4-viruses possess V3 sequences, with an overall positive charge higher than the V3 sequences of R5-viruses. The net charge is calculated by subtracting the number of negatively charged amino acids (D, aspartic acid and E, glutamic acid) from the number of positively charged ones (K and R). In contrast to D and E, which are very similar in their polar and acidic properties, the characteristics of the R guanidinium group differ significantly from the K ammonium group. However, in coreceptor predictive computer algorithms R and K are both equally rated. The study was conducted to analyze differences in infectivity and coreceptor usage because of R-to-K mutations at the V3 positions 9, 10 and 11. V3 loop mutants with all possible RRR-to-KKK triplets were constructed and analyzed for coreceptor usage, infectivity and neutralization by SDF-1α and RANTES. Virus mutants R_9_R_10_R_11_ showed the highest infectivity rates, and were inhibited more efficiently in contrast to the K_9_K_10_K_11_ viruses. They also showed higher efficiency in a virus-gp120 paired infection assay. Especially V3 loop position 9 was relevant for a switch to higher infectivity when occupied by R. Thus, K-to-R exchanges play a role for enhanced viral entry efficiency and should therefore be considered when the viral phenotype is predicted based on V3 sequence data.

## Introduction

The third variable region (V3 loop) of the HIV-1 envelope protein (gp120) plays an important role in HIV-1 infection [1,2], being the primary determinant for binding to one of the two 7-transmembrane receptors CCR5 (R5-tropic) and CXCR4 (X4-tropic) [3,4]. Only because of the V3 loop amino acid sequence, or even because of a single amino acid mutation within the V3 loop, HIV-1 can shift from the R5- to the X4-tropic phenotype [5] or can develop R5X4-dualtropism. Both, CXCR4 and CCR5 are cell membrane associated G-protein-coupled receptors who trigger calcium signaling after binding their ligands, which are SDF-1α (stromal cell-derived factor-1 alpha) for CXCR4 and RANTES (regulated on activation, normal T cell expressed and secreted), Mip-1α and Mip-1β for CCR5. Since chemokines compete with the V3 loop for binding to one of the coreceptors, they can be used to study viral entry efficiencies in virus-neutralization experiments [6,7].

Although the biologically relevant structure of the V3 loop has been identified in its CD4 bound state, the initial steps and their precise mechanism by which the V3 loop binds to CXCR4 or CCR5 is not fully understood as the V3 loop is extremely variable. It is suggested that binding of gp120 to CD4 initiates a structural change of the gp120 trimer [8]. This leads to the exposure of a protruding cluster of three V3 loops all together pointing towards the chemokine receptor [9]. In this state the V3 loop consists of three structural elements. Firstly, a conserved antiparallel strand at the base of the loop which is joined by a Cys-Cys bridged disulfide bond. Secondly, a sequence variable and highly flexible stem region and thirdly, a conserved turn-motif forming the tip of the V3 loop [1].

Besides the three sequence motifs, the overall amount of positively charged amino acids arginine (R) and lysine (K) and negatively charged aspartic (D) and glutamic (E) acids plays an important role for coreceptor usage. From the total number of R, K, D and E amino acids, the overall charge (net charge) is calculated (R+K-D-E) and V3 loop sequences (cysteine-to-cysteine) with a net charge <+4 are commonly associated with R5-tropism. Net charges >+5 are associated with X4-tropism, including the presence of K and R residues at positions 11 and 25 [5,10]. Thus, V3 loop sequence data can be analyzed for R/K and D/E amino acids to differentiate R5- from X4-tropism.

With the availability of coreceptor inhibitors [11], as one part of the armamentarium of anti-HIV-1 drugs, it became important to analyze circulating virus strains for their coreceptor usage [12]. HIV-1 has the general potential to use CXCR4 and/or CCR5 but only the CCR5 pathway can be blocked by medical interventions, so it is important to monitor coreceptor tropism of the most frequent viruses in patients to adapt treatment procedures. Determination of virus tropism by a cell based entry assay using patient isolates or gp120 pseudotyped viruses will provide the best information on coreceptor usage, but it is a time consuming and expensive method that needs special knowledge and laboratory safety equipment. In contrast to such traditional virological methods, computer algorithms have been developed to predict coreceptor usage based only on amino acid sequence data for HIV-1 subtype B [13–20] and subtype C [21] V3 loops.

One of the first rules that relied on V3 sequence data to predict a corresponding coreceptor usage was the 11/25 rule (positions in the V3 loop relevant for coreceptor prediction). This rule refers to two amino acid positions within the V3 loop which are the determinants of HIV-1 tropism [5,10,22]. This coreceptor predictive rule was later extended by Cardozo and coworkers [23] and is known as the 11/24/25 predictive rule. The two bioinformatics tools, geno2pheno [24] and WebPSSM [16], also consider the 11/24/25 rule. In both algorithms, the V3 loop net charge is calculated on the basis of the sum of R and K subtracted by the sum of D and E amino acids and all four amino acids are voted equally for their positive or negative charge. The negatively charged amino acids D and E are very similar and only differ in one -CH_2_- group in their side chain. However, the positively charged amino acids R and K are structurally very different and cannot be easily replaced with each other without significant influence on structure and biological activity of proteins [25–27]. Since the influence of R and K on V3 structure and biological activity might be different, we have studied mutants of HIV-1 for infectivity which contain different R/K motifs within the V3 loop. For the study, HIV-1 viruses with all triple combinations of arginine and lysine at the V3 loop (amino acid positions 9, 10 and 11) were constructed. These positions were chosen since they were often occupied by R and K in virus isolates. Mutants were constructed in the background of the NL-952 virus [28]. The NL-952 virus showed a R_11_G_24_E_25_ (G, glycine) sequence that is a typical indication for R5-tropism but was special since it had a high content of arginine and lysine amino acids causing a V3 net charge of + 5, pointing towards CXCR4 usage. NL-952 also showed the V3 sequence motif R_9_K_10_R_11_ which is another indicator for CXCR4 usage. Thus taken together, the NL-952 virus is R5X4-dualtropic. An interesting additional observation was that the NL-952 mutation NNT>QNT (termed NL-952.2) (N, asparagine; Q, glutamine), leading to the lack of the V3 loop N-glycan g15, caused the shift from R5X4- to X4-tropism. All generated virus mutants were tested for infectivity and neutralization by chemokines and in a cell based competitor assay against purified soluble gp120 [29]. The highest rates for viral entry were seen for the R_9_R_10_R_11_ and the lowest for the K_9_K_10_K_11_ virus mutants.

## Materials and Methods

### Cloning of NL-952 V3 loop mutants

HIV-1 952-env (BstEII-BamHI) plasmids (pUCenv) [28] were used to construct mutants at the 9, 10 and 11 position by site directed mutagenesis using 30mer primers (Metabion, Germany). Polymerase chain reaction was carried out in a total volume of 50 μl containing 3 μl (25 mM) MgCl_2_, 5 μl 10 x PCR-buffer, 4 μl (25 mM) dNTP-Mix, 10 units Pfu-Polymerase (Fermentas, Germany), 5–10 ng plasmid DNA and 2 μl (10 pM) of each of the 30mer primers. The PCR cycles were: 1: 94°C for 30 sec, 2: 62°C for 30sec, 3: 2 min/kb DNA 68°C. Cycles 1–3 were repeated 20 times. The amplified DNA was incubated with 10 units DpnI (Fermentas, Germany) for 2 h at 37°C. Five μl of DpnI treated amplified plasmid DNA was transformed into *E*. *coli* XL1-blue and single colonies were analyzed by env sequencing. The env fragment was cloned via BstEII and BamHI compatible sites into the pNL4-3-Bst [28] virus vector or into the env expression plasmid pSVATGrev as described earlier [30].

### Infection assay

The infectivity of virus mutants was monitored as described by Reed & Muench [31]. Each well of a 96-well plate, containing 10^4^ TZM-bl cells (CD4+, CXCR4+, CCR5+), cultured in Dulbecco's Modified Eagle Medium (DMEM; Gibco, Germany) including 10% fetal calf serum (FCS; Biochrom, Germany), were infected with virus supernatant representing a virus amount of 5 ng p24/ml and was incubated for 48 h. Viral entry into TZM-bl cells causes the expression of β-galactosidase, which was monitored by X-gal (5-bromo-4-chloro-3-indolyl-β-D-galactopyranoside) staining. The cell culture supernatant was removed from the plates and the cell layer was treated with 0.25% glutaraldehyde in phosphate buffered saline (PBS, 5 min, RT) and washed three times with PBS. The PBS was removed and 100 μl of the staining solution (0.5 mg X-gal/ml, 1 mM MgCl_2_, 3 mM potassium ferrocyanide, 3 mM potassium ferricyanide in PBS) was added to each well and the plates were incubated at 37°C for 1 h. After the development of blue stained cells (foci) the plates were washed with PBS and the foci were counted. The specific infectivity for each of the virus mutants was expressed as the number of foci counted (foci forming units, ffu) per amount of virus (ng p24) used for the inoculation of cells (ffu/ng p24).

### Neutralization assay

Cells were infected with a volume of virus containing cell culture supernatant, representing an infectious dose of about 100 ± 20 foci. Virus was added to 10^4^ cells/96-well after pre-incubation of the cells with either SDF-1α (GHOST-X4 cells, CD4+, CXCR4+) or RANTES (GHOST-R5 cells, CD4+, CCR5+) at various concentrations (0, 125, 250, 500 and 1000 ng/ml) for 30 minutes. The cell cultures were incubated at 37°C. After 3 days the 96-well plates were washed two times using PBS and the cell layer was treated with formaldehyde (1% in PBS) for 2 min at RT. The plates were washed two times using PBS and the fixated cells were tested for GFP expression by fluorescence microscopy. To monitor GHOST-cell infection the green foci were counted. In general, GHOST-R5 and GHOST-X4 cells were cultured in DMEM (Gibco, Germany) including 10% FCS (Biochrom, Gemany). In neutralisation experiments using mHSA (modified human serum albumin) and TZM-bl cells, the infection rates of TZM-bl cells were estimated as described above by X-gal staining.

### Soluble gp120

To produce cell culture supernatants which contain soluble gp120 (sgp120), the BstEII/BamHI env fragement of NL-952-RRR and NL-952-KKK was cloned into the BstEII and BamHI compatible sites of the expression vector pSVATGrev as described earlier [30]. HeLa-P4 cells were transfected with pSVATGrev-RRR and pSVATGrev-KKK and cell culture supernatants were harvested. In these supernatants the sgp120 concentration was between 25 and 50 ng/ml. This was measured by a dot blot assay using commercial sgp120 (Protein Sciences, U.S.A.) as a standard [29].

### Virus/gp120 competition assay

The competition assay was carried out as described ealier [29]. In brief, TZM-bl cells, cultured in Dulbecco's Modified Eagle Medium (DMEM, Gibco, Germany) including 5% fetal calf serum (FCS, Biochrom, Germany), were simultaneously infected with virus supernatant representing an equal amount of virus (0.5 ng p24 in 100 μl/96-well) and in the presence of soluble gp120 at a final concentration of 7.5 ng/ml. The plates were incubated at 37°C for 48 h. After X-gal staining, blue foci were counted and the infection rate was expressed as ffu/ng p24. In addition, experiments were carried out with TZM-bl cells preincubated with polybrene at a concentration of 2 μg/ml. The polybrene treatment was carried out at room temperature for 1 h followed by two wash cycles with PBS.

## Results

### Specific infectivity of V3 loop amino acid mutants

**Fig 1:**
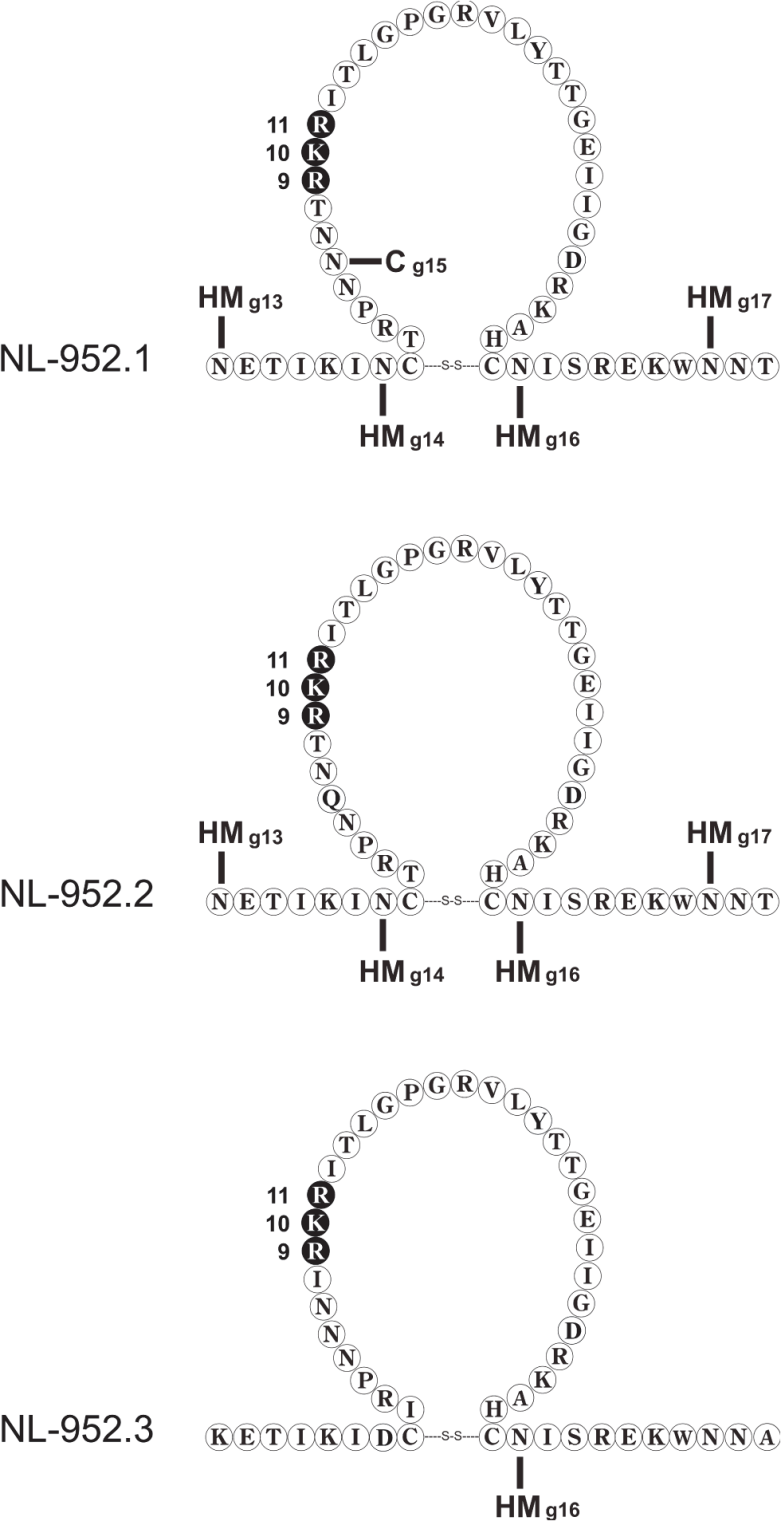
NL-952 virus mutants. V3 loop regions of the three viruses NL-952.1, NL-952.2 and NL-952.3 which differ by mutations affecting the N-glycosylation sites g13-g17. The three viruses were used to generate mutants with all triple combinations of amino acids R and K at the V3 loop positions 9, 10 and 11 (black symbols). N-linked carbohydrates: C, complex type; HM, high mannose type.

**Fig 2:**
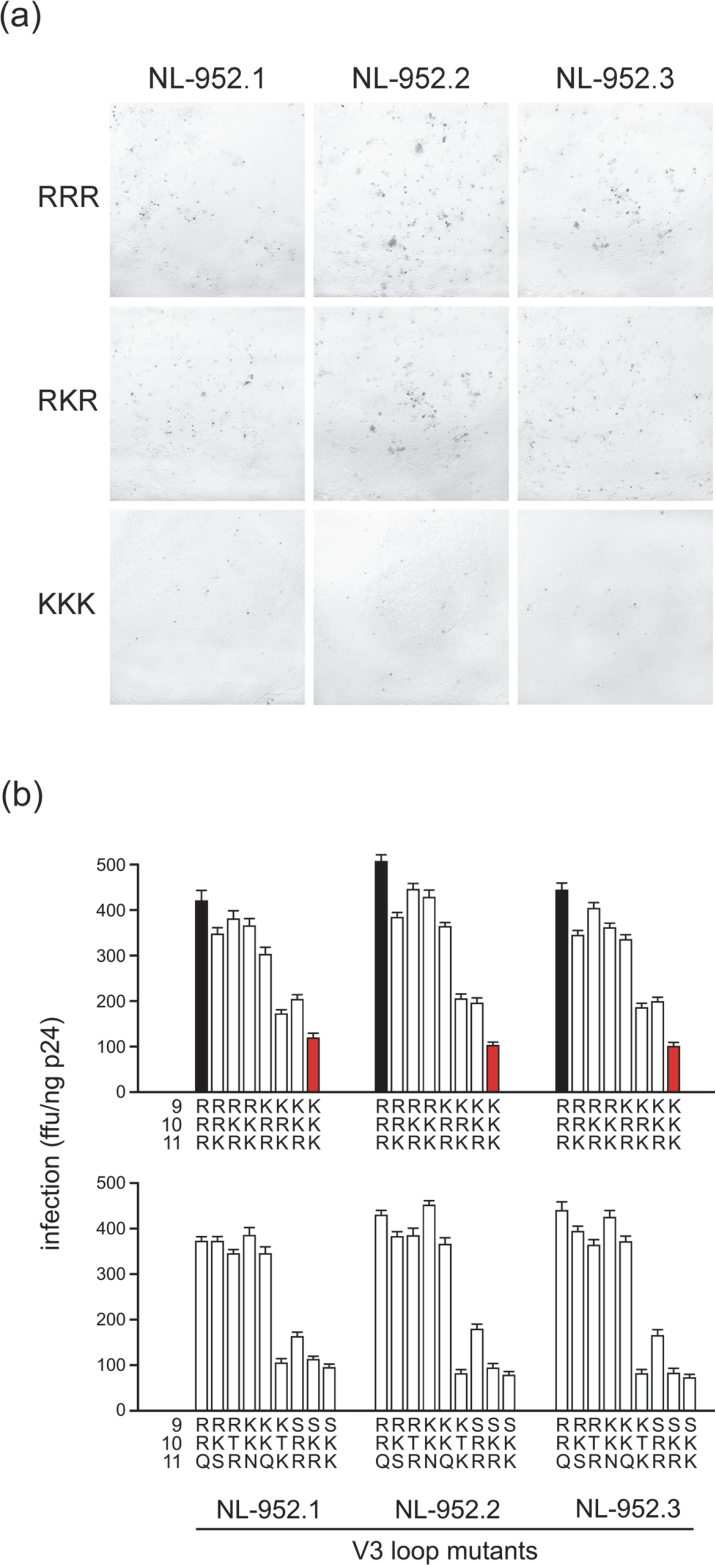
Infection rates of RRR-to-KKK V3 loop mutants. (a) Blue stained TZM-bl cells that had been infected by RRR, RKR and KKK mutants of viruses NL-952.1, NL-952.2 and NL-952.3. For each experiment, about 10^4^ cells/96-well were infected with cell culture supernatants containing virus equal to 0,5 ng p24. Staining with X-gal was carried out two days post infection. (b) Viruses containing the amino acid combinations as shown in Table 1 at the V3 loop positions 9-to-11 were tested for infectivity using CD4+, CCR5+ and CXCR4+ TZM-bl cells. Shown are the means and standard deviations based on ten experiments. Virus inocula were standardized based on p24 antigen measurements (0,5 ng p24/10^4^ cells/96-well). Within the set of RRR-to-KKK mutants, all RRR-virus mutants (black bars) showed the highest and the KKK-virus mutants (red bars) the lowest infection rates.

A panel of virus mutants with all triple combinations of amino acids R and K at the V3 loop positions 9, 10 and/or 11 was constructed in the background of the NL-952 virus, designated RRR-to-KKK mutants. As a viral background we used three different NL-952 constructs designated NL-952.1, NL-952.2 and NL-952.3. The three NL-952 constructs differ in the V3 region, especially at the sites for N-glycosylation g13, g14, g15 and g17 (Fig. 1). The V3 loop with the R_9_K_10_R_11_ amino acid motif was originally from a R5X4-tropic PI-952 patient isolate and in the NL4-3 context the PI-952 V3 loop also showed R5X4-dualtropism. Interestingly, the single amino acid exchange to RRR (RKR>RRR) caused the loss of CCR5 usage, changing the virus to X4-monotropism. The same loss of CCR5 usage was seen for the two NL-952 constructs NL-952.2 and NL-952.3. For these two viruses, all corresponding RRR-to-KKK mutants showed X4-monotropism and we have observed no replication of any of these 16 mutants in GHOST-R5 cells. Thus, CCR5 usage was lost due to the lack of N-glycans in the V3 loop and even a KKK mutant showed no replication in GHOST-R5 cells (Table 1). As shown in Fig. 2A exemplarily for RRR, RKR and KKK and for all mutants in Fig. 2B, the infectivity rates of all three sets of RRR- to KKK-mutants were reduced in a descending order from (i) the RRR- to the (RRK, RKR, RKK, KRR)-mutants followed by (ii) the (KRK, KKR) mutants and finally (iii) by the KKK-mutants which had the lowest infection rates. In addition to the RRR-to-KKK mutants we have constructed a second set of 27 mutants with N, Q, S and T amino acids changes at position 9-to-11. These four amino acids are frequently present in the 9-to-11 region according to a V3 loop sequence alignment of subtype B provided by the Los Alamos data base [32]. For these NQST controls, infectivity rates were significantly different between the (RRQ, RKS, RTR, KKN, KKQ)- and (KTK, SRR, SKR, SKK)-group, (S, serine; Fig. 2B). The specific infectivity of these mutants was tested in TZM-bl cells as shown in Fig. 2B and their coreceptor usage was tested in GHOST-X4 and GHOST-R5 cells. Infectivity expressed as ffu/ng p24 was measured 72 h post infection by counting infected cells and by comparing these results with the amount of p24 that was used for infection. Again, the exchange of amino acid R (R>K, R>S) at position 9 reduced the infectivity to 20–30% when compared to the RRQ or RRR mutants. Additionally, all 27 mutants showed R5-monotropism despite the presence of the R amino acid at position 9 (RRQ, RKS, RTR) (T, threonine; Table 1). Thus, only two positively charged amino acids in the 9-to-11 region were not sufficient to allow CXCR4 usage and the presence or lack of the N-glycan g15 was therefore without influence on coreceptor usage.

**Table 1:** Coreceptor usage and infectivity of NL-952 V3 loop mutants.

V3 loop Position 9, 10, 11	virus coreceptor usage and TCID_50_					
	NL-952.1		NL-952.2		NL-952.3	
RRR	X4	4,6 x 10^4^	X4	6,6 x 10^4^	X4	5,1 x 10^4^
RRK	R5X4	3,4 x 10^4^	X4	4,6 x 10^4^	X4	3,3 x 10^4^
RKR	R5X4	3,9 x 10^4^	X4	5,0 x 10^4^	X4	4,1 x 10^4^
RKK	R5X4	3,2 x 10^4^	X4	3,3 x 10^4^	X4	3,4 x 10^4^
KRR	R5X4	2,6 x 10^4^	X4	3,5 x 10^4^	X4	3,0 x 10^4^
KRK	R5X4	9,1 x 10^3^	X4	9,1 x 10^3^	X4	8,8 x 10^3^
KKR	R5X4	8,4 x 10^3^	X4	9,1 x 10^3^	X4	8,8 x 10^3^
KKK	R5X4	7,0 x 10^3^	X4	6,6 x 10^3^	X4	6,6 x 10^3^
RRQ	R5	3,6 x 10^4^	R5	5,3 x 10^4^	R5	5,2 x 10^4^
RKS	R5	3,5 x 10^4^	R5	4,9 x 10^4^	R5	4,0 x 10^4^
RTR	R5	2,5 x 10^4^	R5	3,4 x 10^4^	R5	3,2 x 10^4^
KKN	R5	3,0 x 10^4^	R5	3,1 x 10^4^	R5	4,0 x 10^4^
KKQ	R5	2,6 x 10^4^	R5	3,5 x 10^4^	R5	3,8 x 10^4^
KTK	R5	6,3 x 10^3^	R5	6,5 x 10^3^	R5	6,1 x 10^3^
SRR	R5	8,0 x 10^3^	R5	8,7 x 10^3^	R5	8,1 x 10^3^
SKR	R5	6,5 x 10^3^	R5	6,6 x 10^3^	R5	6,1 x 10^3^
SKK	R5	6,0 x 10^3^	R5	6,1 x 10^3^	R5	5,9 x 10^3^

As shown in Fig. 2B, virus mutants containing RRR or RRQ showed the highest specific infectivity and the KKK, SKK mutants the lowest infectivity rates. Thus, arginine at position 9 was most important for enhanced NL-952 infectivity. A RKK>KKK change caused a 60% loss of infectivity which was also seen for the RKK>SKK mutant. Infectivity decreased from RRR>KRR>SRR indicating that the loss of the positive charge at position 9 had the highest impact on infectivity but not on coreceptor usage principally.

### Effect of amino acids R and K on resistance to SDF-1α, RANTES and mHSA

**Fig 3:**
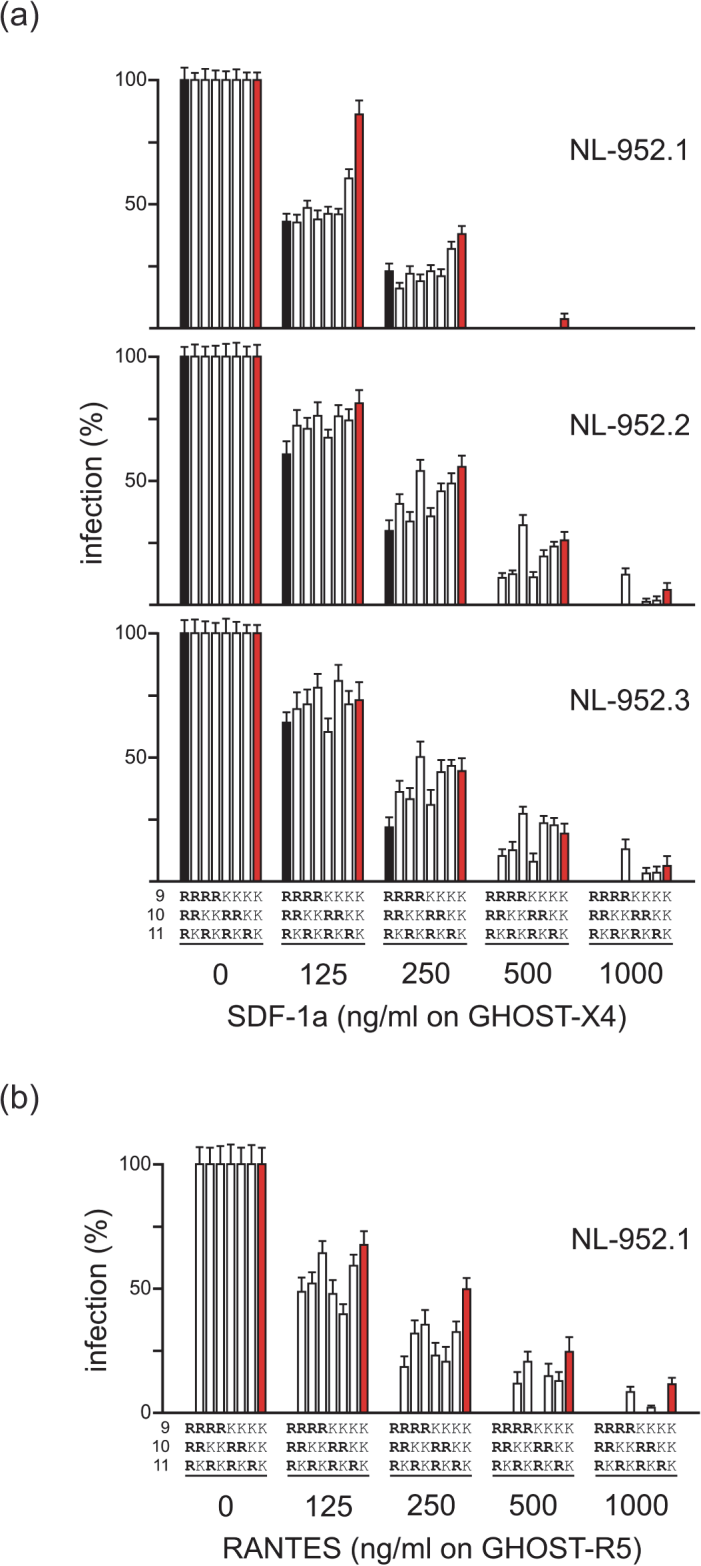
Neutralization of RRR-to-KKK V3 loop mutants by SDF-1α, RANTES. (a) For each NL-952.1, NL-952.2 and NL-952.3 virus all eight mutants (RRR-to-KKK) were tested for neutralization by SDF-1α. GHOST-X4 cells were infected with an amount of virus representing about 100 ffu. SDF-1α was added to a final concentration of 125, 250, 500 and 1000 ng/ml. (b) Neutralization by RANTES was carried out for X4R5-dualtropic NL-952.1 mutants with the exception of the X4-monotropic mutant RRR, since the RRR mutant does not show any viral growth in GHOST-R5 cells (infection = 0% at RANTES 0 ng/ml). Also all NL-952.2 and NL-952.3 mutants did not replicate in GHOST-R5 cells and therefore could not be tested for neutralization by RANTES.

**Fig 4:**
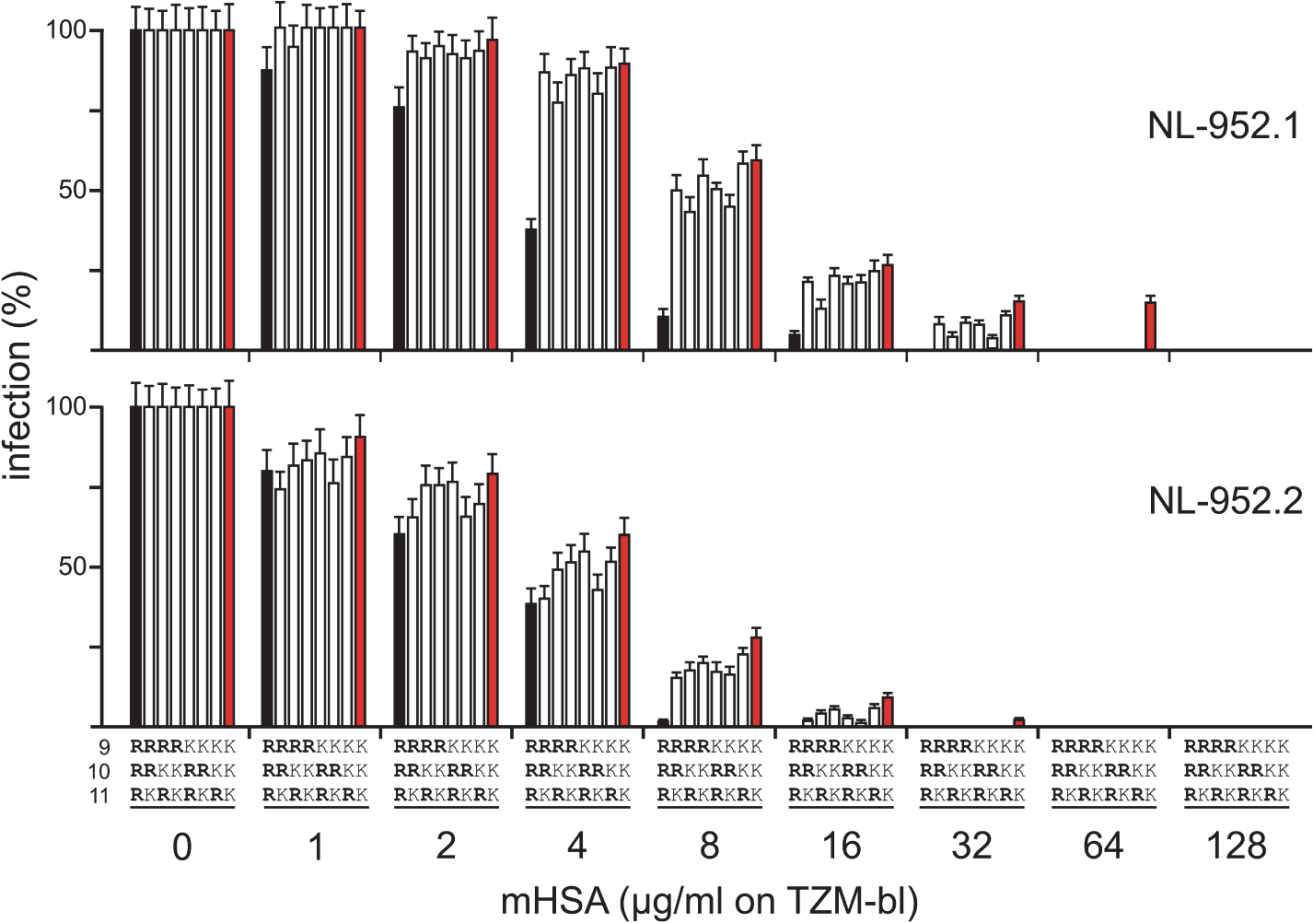
Neutralization of RRR-to-KKK V3 loop mutants by mHSA. The eight RRR-to-KKK mutants were tested for neutralization against the HIV-1 inhibitory protein mHSA [30]. TZM-bl cells were infected with an amount of virus representing about 100 ffu. The HIV-1 entry inhibitor mHSA was added at various concentrations. Shown are the means and standard deviations based on ten experiments. Black bars, RRR-mutants; red bar, KKK-mutants.

The RRR-to-KKK X4, R5 and R5X4-tropic mutants were tested in neutralization assays against SDF-1α (GHOST-X4 cells) (Fig. 3A), RANTES (GHOST-R5 cells) (Fig. 3B) and mHSA (TZM-bl cells) (Fig. 4) according to their coreceptor type. Virus neutralization was measured 3 days post infection by counting infected cells. As shown in Fig. 3A all virus mutants of the NL-952.1, but not the KKK, virus showed a comparable sensitivity against SDF-1α at 125 and 250 ng/ml. The NL-952.1-KKK virus showed a low replication rate of ≈5% at 500 ng/ml. At this concentration, all the other mutants were completely neutralized. At 1000 ng SDF-1α per ml all mutants were neutralized with no exception. Interestingly, the lack of N-glycan g15 altered the KKK, KKR and KRK mutants to be more resistant against SDF-1α. The mutant with the amino acid motif RKK was the most resistant virus in the panel of the −g15 mutants of the NL–952.2 and NL–952.3 viruses. The same trend of resistance was observed in the RANTES neutralization experiments (Fig. 3B). Here RKK, KRK and the KKK mutant of NL-952.1 showed the highest resistance against RANTES, and these viruses showed replication at 1000 ng/ml. The most RANTES sensitive mutants were RRK and KRR which were completely neutralized by 500 ng RANTES/ml. Using the HIV-1 inhibitor mHSA and TZM-bl indicator cells (Fig. 4) the results for neutralization by SDF-1α and RANTES were confirmed. Again, the two arginine containing viruses (RRK, KRR, RKR) that showed high infectivity rates per ng p24 were neutralized more efficiently compared to the slower replicating KKK mutants. Thus, the slow KKK mutants were the most resistant viruses in that panel of mutants and vice versa.

**Fig 5:**
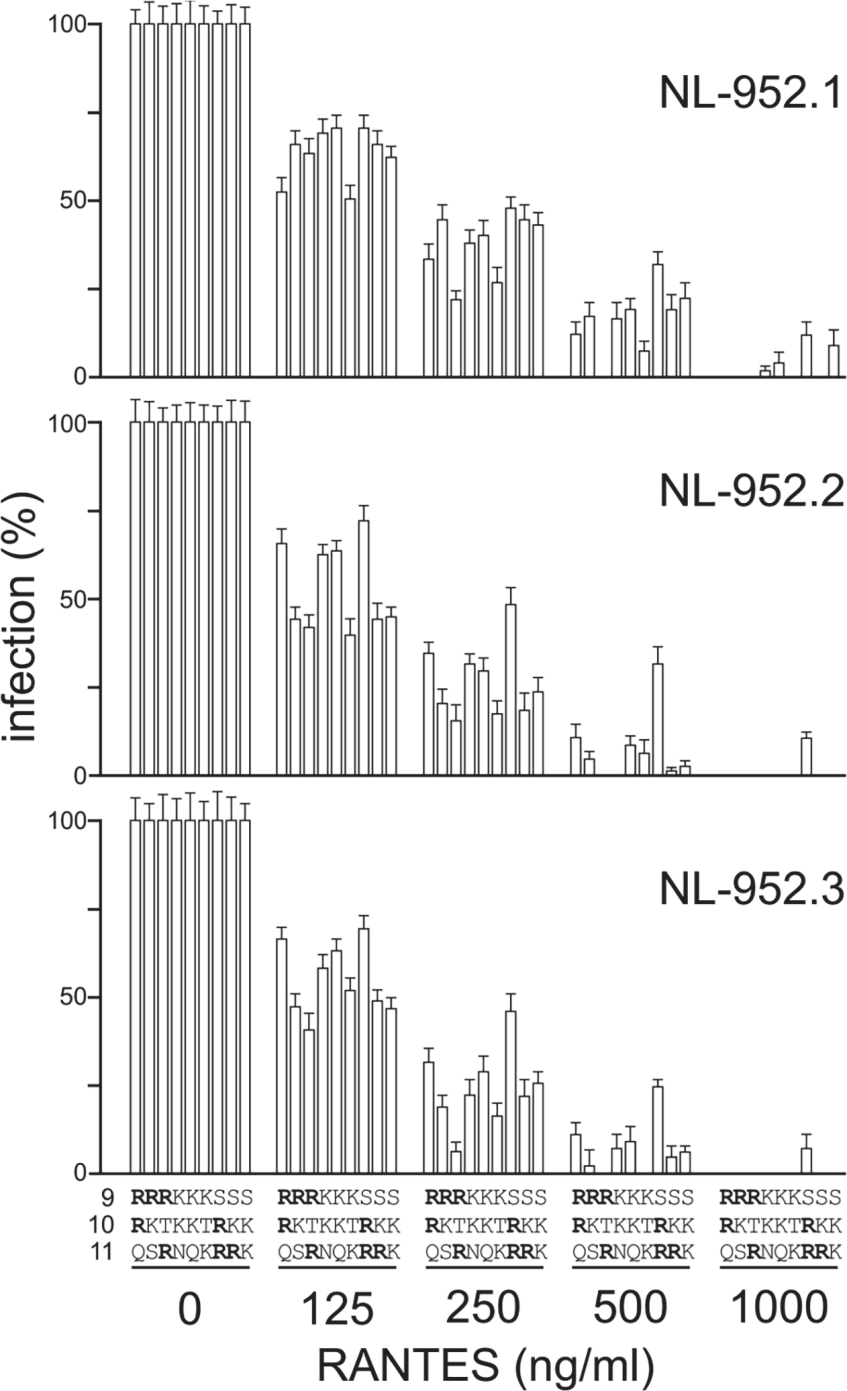
Neutralization of NQST- V3 loop mutants by RANTES. For each of the three viruses NL-952.1, NL-952.2 and NL-952.3, nine mutants containing amino acids N, Q, S and T at the V3 position 9, 10 and/or 11 were tested for neutralization by RANTES. GHOST-R5 cells were infected with an amount of virus representing about 100 ffu. RANTES was added to a final concentration of 125, 250, 500 and 1000 ng/ml. All 27 viruses were R5-monotropic since they showed no growth on GHOST-X4 cells (see also Table 1). Shown are the means and standard deviations based on ten experiments.

In addition we have constructed a set of V3 mutants which contain R and K residues but also residues of frequently present amino acids in the 9-to-11 region: N, Q, S and T [32] (Fig. 5). Due to the exchange of R or K against one of these amino acids all nine mutants switched to R5-monotropism and therefore, coreceptor-specific neutralization could only be tested against the chemokine RANTES. The data showed that the X4-coreceptor specificity of the NL-952.1-RRR mutant was changed to R5-monotropism by a single amino acid mutation RRR>SRR. Additionally, the SRR mutant showed the highest level of RANTES resistance that was not influenced by lack or presence of the N-glycan g15. At 250 and 500 ng RANTES/ml the SRR mutants with R>K exchanges, SKR and SKK showed −g15 dependent RANTES sensitivity when compared to the +g15 mutants. Thus, the effect caused by the lack of g15 was not compensated by R>K exchanges. The highest levels of sensitivity against RANTES were observed when R_10_ or K_10_ was replaced by threonine (RTR, KTK). These two mutants were neutralized most efficiently and we have seen no effects caused by mutations affecting the g15 N-glycosylation sites or because of the R and K amino acids at position 9 and 11. In agreement with the RRR>KKK mutants, the replacement of one of the R or K amino acids by Q (RRQ, KKQ), T (RTR, KTK) or S (SRR, SKK) changed the viral coreceptor phenotype from X4- or R5X4- tropism to R5-monotropism, respectively. Thus, R5X4-dualtropism of the NL952.1 virus was only seen with all R/K combinations (despite RRR, X4-tropic) at the V3 loop positions 9-to-11. An overall general tendency, that the viruses with the highest replication rates were more sensitive to neutralization in contrast to the low replicating viruses as it was shown in the SDF-1α experiments (Fig. 3A) could not be confirmed for CCR5-specific infection.

### Coreceptor switch and V3 loop N-glycosylation

The NL-952.2 and NL-952.3 viruses are both lacking N-glycosylation sites within and next to the V3 loop (Fig. 1). The presence of N-glycan g15 within the V3 loop of the RRR-to-KKK mutants was shown to be correlated to the R5X4-dualtropic phenotype with the exception of the triple R mutant NL-952.1-RRR. For the X4-to-R5X4 shift a single switch from R-to-K was sufficient at any of the three positions (RRR>KRR, RRR>RKR and RRR>RRK). The dualtropic phenotype was shifted to X4-monotropism by the mutation in the N-glycosylation site for g15 (NNT>QNT) as a sufficient condition. On the other hand the R5 phenotype was shifted to X4 RRQ>RRK, RTR>RKR and SRR>KRR, thus K at positions 9, 10 or 11 had the same influence on R5-to-X4 switch in viruses lacking N-glycan g15. In contrast, in NL-952.1 mutants KRR, RKR and RRK containing g15, an accumulation of the CXCR4 usage was observed (R5-to-R5X4 switch). N-glycan g15 seems to mask positively charged amino acids since the lack of g15 leads to X4-monotropism in the RRK-to-KKK +5 charged V3 loop mutants. The NQST-mutants with an overall positive charge of +4 all showed the R5 phenotype. The R5 phenotype of NQST-mutants was observed regardless whether N-glycan g15 was present or not. Moreover, the R5 phenotype of NQST-mutants was shifted back to X4-monotropism by introducing R or K at positions 9, 10 or 11 in the background of the NL-952.2 and NL-952.3 virus.

### Viral infectivity in the presence of soluble gp120

**Fig 6:**
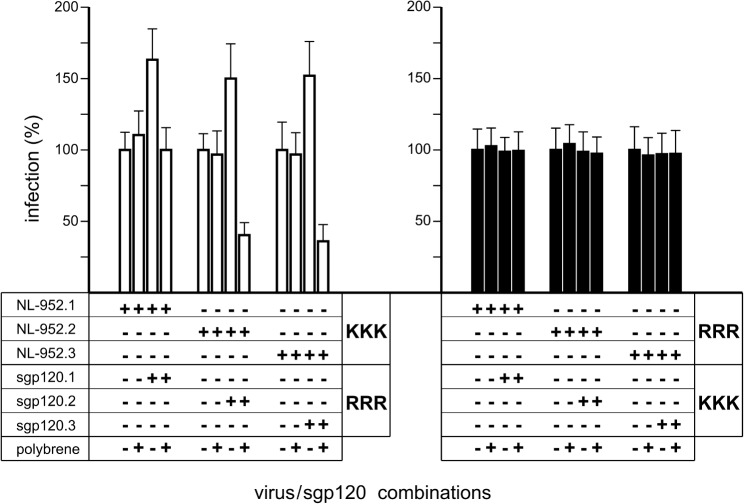
Impact of soluble gp120 on viral entry. For each experiment, TZM-bl cells were infected with cell culture supernatants containing virus equal to 0,5 ng p24 antigen in a volume of 100 μl and in the presence of 7,5 ng sgp120/ml. Soluble gp120 was produced in HeLa-P4 cells using pSVATGrev expression vectors for sgp120-RRR and sgp120-KKK. White bars, experiments with KKK-viruses and RRR-sgp120. Black bars, experiments with RRR-viruses and KKK-sgp120. +, indicate the component parts of each virus/sgp120 experiment. Shown are the means and standard deviations based on three experiments.

We have further studied the influence of soluble gp120 on viral entry (Fig. 6) using an assay that allows monitoring infection in relation to another virus envelope without direct replicating rivalry by a second virus. Therefore, we have generated the three different glycosylated forms of gp120 as RRR and KKK mutants (sgp120.1-RRR, sgp120.2-RRR, sgp120.3-RRR, sgp120.1-KKK, sgp120.2-KKK, sgp120.3-KKK) in HeLa-P4 cells. For example, cells transfected with pSVATGrev.1-RRR released sgp120.1-RRR into the cell culture medium. These gp120-containing supernatants were used and a volume representing 7.5 ng was mixed with a virus supernatant representing a viral dose of 5 ng p24 into a final volume of 1 ml culture medium. After preincubation, 100 μl of this mixture was added to each well of a 96-well microtiter plate containing 10^4^ TZM-bl cells per well.

The infectivity of all three RRR-mutants (Fig. 6; black bars) was not influenced by soluble gp120 of the KKK type. Also no enhancing or neutralizing effect was observed when the TZM-bl cells were pretreated with polybrene (2 μg/ml). Polybrene is a cationic polymer that is masking negatively charge molecules like heparan sulfates and sialic acid present on the cell surface generally used by X4-tropic viruses as adhesion molecules. A more significant effect on viral infectivity was observed with the combination of the neutralization resistant but slow virus mutants of the KKK type and the sgp120 corresponding to the fast RRR-type (Fig. 6; white bars). All three KKK mutants showed enhanced infectivity when the RRR sgp120 was added. This effect was not seen with polybrene pretreated cells. Here the sgp120-RRR, corresponding to the faster replicating phenotype, was reducing the infectivity of the virus mutants lacking N-glycans. In contrast, the combination of the fully glycosylated form of sgp120 and its viral counterpart showed no effect after polybrene treatment. The data showed that the enhancing effect was independent of the presence of N-glycans, whereas the neutralizing effect was only seen for virus and sgp120 with a lack of N-glycans and after polybrene treatment.

### Prediction of coreceptor usage for NL-952 V3 loop mutants

We have compared our results on coreceptor usage with the bioinformatics tools geno2pheno, PSSM_R5X4_ PSSM_SINI_ and HIVcoPred all freely available on the World Wide Web (Table 2). The best coincidence was observed for the set of NL-952.2 viruses of the RRR-to-KKK mutants that all showed the X4-monotropic phenotype. Here, all algorithms correctly predicted the X4-type. In contrast to the set of NQST mutants which were R5-monotropic, all the algorithms incorrectly predicted X4-monotropism. The prognosis for the set of NL-952.1 mutants was more complex. The hit ratio was low with 2/17 for HIVcoPred, 3/17 for PSSM_X4R5_, 4/17 for geno2pheno and 5/17 for PSSM_SINI_. The best result was obtained for the RRR mutants. Here, all algorithms predicted correctly X4 but the V3 sequence from the original PI-952 isolate with the RKR motif was not predicted correctly by any of the algorithms. This was also observed for all other R5X4 viruses that were prognosed X4. The R5 viruses were analyzed best by the PSSM_SINI_ algorithm. Here, four out of nine R5 viruses were predicted correctly. Especially for the low replicating, neutralization resistant viruses KTK, SRR, SKR and SKK the prediction was X4, but all viruses showed the R5 monotropic phenotype.

**Table 2:** Coreceptor prediction for NL-952.1, NL-952.2 and NL-952.3 V3 loop mutants.

Virus	NL-952.1					NL-952.2 and NL-952.3				
	CC	G2P	PSSM		HcP	CC	G2P	PSSM		HcP
			SINSI	R5X4				SINSI	R5X4	
RRR*	**X4**	**X4**	**X4**	**X4**	**X4**	**X4**	**X4**	**X4**	**X4**	**X4**
RRK	X4R5	X4	X4	X4	X4	**X4**	**X4**	**X4**	**X4**	**X4**
RKR	X4R5	X4	X4	X4	X4	**X4**	**X4**	**X4**	**X4**	**X4**
RKK	X4R5	X4	X4	X4	X4	**X4**	**X4**	**X4**	**X4**	**X4**
KRR	X4R5	X4	X4	X4	X4	**X4**	**X4**	**X4**	**X4**	**X4**
KRK	X4R5	X4	X4	X4	X4	**X4**	**X4**	**X4**	**X4**	**X4**
KKR	X4R5	X4	X4	X4	X4	**X4**	**X4**	**X4**	**X4**	**X4**
KKK	X4R5	X4	X4	X4	X4	**X4**	**X4**	**X4**	**X4**	**X4**
RRQ	**R5**	X4	**R5**	**R5**	X4	R5	X4	X4	X4	X4
RKS	**R5**	**R5**	**R5**	**R5**	**R5**	R5	X4	X4	X4	X4
RTR	R5	X4	X4	X4	X4	R5	X4	X4	X4	X4
KKN	**R5**	**R5**	**R5**	X4	X4	R5	X4	X4	X4	X4
KKQ	**R5**	**R5**	**R5**	X4	X4	R5	X4	X4	X4	X4
KTK	R5	X4	X4	X4	X4	R5	X4	X4	X4	X4
SRR	R5	X4	X4	X4	X4	R5	X4	X4	X4	X4
SKR	R5	X4	X4	X4	X4	R5	X4	X4	X4	X4
SKK	R5	X4	X4	X4	X4	R5	X4	X4	X4	X4
Predicted correctly		4	5	3	2		8	8	8	8

* amino acid sequence at V3 loop positions 9-to-11.

CC = cell culture tested coreceptor usage using GHOST-CXCR4 and GHOST-CCR5 indicator cell lines.

G2P = prediction algorithm geno2pheno (http://www.geno2pheno.org).

PSSM = position-specific scoring matrices (indra.mullins.microbiol.washington.edu/webpssm).

HcP = prediction algorithm HIVcoPred (http://www.imtech.res.in/raghava/hivcopred).

R5X4 = prediction algorithm developed using V3 sequences were CCR5 and CXCR4 coreceptor usage was assayed [14].

SINSI = prediction algorithm developed using V3 sequences were MT2 cell tropism was assayed [14].

## Discussion

### Infectivity and neutralization

In the present study we have constructed and analyzed HIV-1 V3 loop mutants based on a V3 loop from the patient isolate PI-952. At the V3 loop amino acid positions 9-to-11 the PI-952 isolate showed three positively charged amino acids RKR, showed R5X4-dualtropism and was resistant to neutralization. A well accepted method to study V3 loop dependent viral entry is the transfer of the V3 sequence from a patient isolate into the background of i.e. the NL4-3 virus [28,33]. Therefore, we also introduced the PI-952 V3 sequence into the NL4-3 virus and obtained a NL-952 chimera which showed the original R5X4-dualtropic phenotype of the patient isolate and was also resistant to neutralization as much as the original PI-952 isolate. Other known HIV-1 strains or isolates with similar amino acid sequences at the 9-to-11 position are only X4-monotropic as for example the virus strains HXB2, ADAV3B (RKR, [34]), HTLVIIIB (RKK, [35]), KF6 (KKR, [36]) and T16 (RRR, [36]). A statistical sequence analysis by Hung et al. [37] of V3 loops with known coreceptor usage revealed that for R5-tropism the 9-to-11 sequence is mainly occupied by R_100_(K_80_R_4_)S_88_, for R5X4-tropism by R_87_(K_87_R_13_)(R_50_ S_50_) and for X4-tropism by R_90_(K_40_R_20_)(R_50_S_30_K_20_). The increase in the positively charged amino acid arginine in this region seems to be critical to shift the virus from R5- to the X4-tropism. Data from Hung et al. [37] also showed that the insertion of arginine instead of lysine, and the loss of the N-linked glycosylation site g15 in the V3 loop region are both strong correlates for X4-tropism. Our data on the +/− N-glycosylated RRR>KKK mutants are in agreement with other studies which also showed an important role for arginine and the lack of N-glycan g15 for X4-tropism [36,38,39]. In NL-952.2 and NL-952.3 the lack of N-glycan g15 clearly caused the X4 shift, whereas the lack of one of the positively charged amino acids R or K at any of the three positions 9, 10 or 11 caused the shift to R5-monotropism.

The patient isolate PI-952 was originally isolated from patient blood as it escaped from autologous neutralization. When we studied virus escape we observed that during disease progression viruses emerge, not neutralized by serum antibody anymore but also harboring mutations effecting N-glycosylation sites [40,41]. We observed that in the late stages of the disease when the immune system becomes selectively impaired [42] these viruses can unmask their gp120 structure to enhance their entry efficiency [33,43]. By presenting an unmasked V3 loop the virus was also more sensitive to neutralization [28]. These observations were in agreement with a study where an increased sensitivity to a broadly neutralizing monoclonal antibody was observed directed against viruses isolated from the late stage of HIV-infection. This effect was due to gp120 unmasking and the accumulation of positive V3 charges [44]. In our present study we showed that NL-952 mutants with amino acid R at the V3 position 9 had the highest infectivity rates and the mutants with K the lowest. The same was observed with virus mutants harboring the N, Q, S, and T amino acid replacements. Again, the 9R mutants showed the highest infectivity rates and the mutants with 9K significantly lower rates. Thus, despite an unchanged, constant V3 net charge, a replacement of amino acid R against K at the V3 loop position 9 has a booster effect on entry efficiency. On the other hand neutralization of the virus mutants revealed that those mutants who showed the lowest replication rates had the highest resistance to neutralization by all tested entry inhibitors and vice versa. At a first glance this seems to be contradictory, but the V3 loop of more efficient viruses might match more precisely to the binding site on their respective coreceptors in contrast to the V3 loop of less efficient viruses. A perfect V3 match, for example to the CCR5 RANTES binding site, also suggests that RANTES will be a more effective inhibitor. Since the −g15 NL-952 mutants switched all to X4-monotropism they could not be tested against RANTES and therefore the influence of g15 on neutralization could not be tested for CCR5-specific infection. However, the lack of N-glycan g15 is found in V3 sequences correlated with X4-tropism more often than in R5-viruses [36,40,45]. This supports other studies which argue that the presence of N-glycan g15 is an important prerequisite for a much better V3 binding to CCR5 [46–48]. These observations are in agreement with our data of the dualtropic NL-952.1 viruses. N-glycan g15 seems to be able to mask positively charged amino acids to allow CCR5 contact as long as the arginine amount is not over a certain limit. In our experiments RRR was not masked sufficiently to be R5X4-tropic but with one arginine less all mutants were R5X4. We hypothesize that the RRR motif with its three arginine residues is not masked by N-glycan g15 in such a way that CCR5 contact can be established and therefore the virus is X4-monotropic. When the coverage of positively charged amino acids is absent, due to the lack of N-glycan g15, the positively residues are free to be exposed and therefore CCR5 contact will no longer be possible. This model would explain why the NL.952.2 and NL-952.3 RRR>KKK viruses can replicate on GHOST-X4 as well as on the CCR5+ and CXCR4+ TZM-bl cells, a HeLa derived cell line. TZM-bl cells are used for viral kinetics and neutralization experiments since they are highly susceptible to HIV-1 and infection is possible via both coreceptors [49]. This is one of the reasons why the X4-monotropic NL-952.2 and NL-952.3 viruses can grow on TZM-bl but not on GHOST-R5.

In contrast to the supporting role of N-glycan g15 for CCR5-specific infection, its presence interferes with CXCR4-specific infection [28,33]. This opposing feature of g15 was also observed when the RRR>KKK mutants were tested against SDF-1α. Mutants of NL-952.2 and NL-952.3, despite NL-952.1-KKK, lacking g15 were more resistant to SDF-1α and also soluble RRR gp120 lacking g15 was neutralizing the KKK viruses. When g15 was present, soluble gp120 could enhance infection in the absence of polybrene but was not neutralizing the NL-952.1-KKK virus when cell surface molecules were masked by polybrene. These experiments support the theory that N-glycan g15 is partly blocking CXCR4-specific infection and that the V3 loop best-fit for making contact to CXCR4 is a sequence lacking the g15 N-glycosylation site N_6_N_7_T_8_.

As mentioned before, one important factor to prevent the virus from binding to a V3-specific neutralizing antibody is the g15 N-glycan within the V3 loop. The observation that an entry efficient virus is highly sensitive to neutralization was also seen in the context of V3 loop glycosylation [28,50]. The lack of g15 leads to higher infectivity [33] but these viruses are prone to neutralization as the unshielded V3 loop is more easily accessible for neutralizing antibody [51]. In our study we have used the gp120 binding inhibitor mHSA [30] instead of antibody and used TZM-bl cells that allow replication of X4-, R5 as well as R5X4-dualtropic strains. This also showed that the −g15 mutants were more sensitive to neutralization compared to the +g15 ones. Thus, the more accessible gp120 V3 loop of the −g15 viruses was a much better target for such an inhibitor. Despite the difference seen in neutralization between +g15 and −g15 mutants, the RRR mutants were more sensitive to neutralization compared to the KKK mutants, which was in agreement with the RANTES and SDF-1α neutralization experiments.

### Effects of soluble gp120 on infection

To study viral entry efficiency of the RRR>KKK mutants, we have used an assay that allows monitoring infection in relation to another virus envelope were its viral replication is not influenced by replicating rivalry of the virus the soluble gp120 belongs to. For this assay we used cell culture supernatants that were harvested from gp120 expressing cell lines. One reason to use soluble gp120 was that it is naturally released from virus particles and can be detected in serum and cell culture fluids [52]. Soluble gp120 was found in blood as well as in lymphoid tissues in significant amounts [53–56]. Depending on the study, the concentrations of soluble gp120 varied between 0.2 and 2.4 ng/ml [55] and between 12 and 96 ng/ml [54]. In our study a concentration of 7.5 ng/ml of soluble gp120 was sufficient to neutralize or enhance HIV-1 entry. In principle, one of the first steps in the process of viral entry is the binding of viral particles to attachment factors on the target cell. If virus attachment is supported, viral entry might be enhanced and obviously a deteriorated attachment will lead to impeded infection.

Besides the major cell surface receptor CD4, syndecans [57] and HSPG [58] are playing an important role for virus attachment and therefore strongly influence HIV-1 infection. Although our experiments were limited to only two gp120-virus combinations, the study unveils that the RRR-envelope with its higher efficiency dominates over the KKK envelope with its lower efficiency, indicating that V3 loop R residues play an important role in binding to cell surface factors.

Peptide studies with R and K homopolymers showed that polymers of six or more R amino acids entered cells more effectively than the K ones [59]. This indicates that the headgroup of R is responsible for the biological activity of these peptides. A more efficient membrane translocation was also shown to be highly dependent on the presence of either heparan sulfates or glycosaminoglycans on the cellular surface [60]. Thus, the R guainidinium groups interact strongly and specifically with anionic moieties that are exposed on complex carbohydrate structures. The HIV-1 accessory factor Tat is a 86 amino acid polypeptide that can bind and pass through membranes easily because of the basic Tat_49–57_ domain RKKRRQRRR. Interestingly, the replacement of K against R demonstrated an enhanced cellular uptake of the Tat_49–57_ peptide when tested as a molecular transporter [61]. These classes of R-enriched peptides, containing the so-called protein transduction domain, bind to HSPG to accumulate on the cell surface before they get internalized by endocytosis [62].

In our study we have masked negatively charged residues on the cellular surface using the polycation polybrene that inhibited the enhancing effect of the soluble RRR-gp120 and promoted the neutralization effect. The same effect was identified when cells were treated with heparinase I to cut off negatively charged HSPG structures, leaving the glycosylated CD4, CCR5 and CXCR4 coreceptor molecules untouched. We hypothesize therefore that V3 loop R amino acids at the 9-to-11 position support interaction with negatively charged groups on the cell membrane that leads to an enhancement of the KKK-viruses. When the HSPG molecules are cut-off by heparinase I or covered by polybrene, the direct coreceptor rivalry is the predominant effect that leads to the suppression of the KKK-gp120 by RRR-gp120 which had a higher entry efficiency.

### Predictive models for coreceptor usage

Normally, the amino acids R and K are of the same value when the positive charge of the V3 loop is calculated to predict virus coreceptor usage. Therefore all software tools used have erroneously predicted the X4-monotropic phenotype which was only accurately calculated for the RRR mutants. The prediction of coreceptor usage by bioinformatics methods is mainly based on the V3 loop amino acid sequence (Cys-Cys) of subtype B [16,63] or subtype C [21] viruses. The methods have become complex but the 11/25 rule or the overall V3 net charge is still an important component of these algorithms. Linking just the two simple elements, 11/25 rule and V3 net charge, the R4-tropism can be predicted assuredly in < 50% but the accuracy for the forecast of R5-tropism is > 90% [64]. The 11/25 rule on its own was proposed to predict for X4 when both positions are occupied by positively charged amino acid residues. Here in our study we have shown that besides its positive charge it should be considered which of the amino acids, K or R, is present in the V3 loop sequence. Especially in context with the N-glycan g15 it seems to be complicated for the predictive algorithms to make reliable forecasts for mutants harboring CCR5 in addition to CXCR4 usage. The correct prediction of the R5X4-dualtropism of the patient derived PI-952 V3 sequence, that includes the RKR motif, was not possible with the G2P, PSSM and HcP computer algorithms. The R5X4-misinterpretation was also observed with all other R/K combinations and only the RRR mutant, which was of X4-type, was predicted correctly independent of the presence or lack of N-glycan g15. Thus, for the NL-952.1 mutants with an +5 overall net charge a change from R to K seems to be not considered in acquiring CCR5 usage. Altogether, the data showed in our study that NL-952 X4-monotropsim can be augured with higher precision than the R5X4-dualtropic or R5-tropic phenotype. In the NL-952.2 background lacking N-glycan g15, all RRR-to-KKK +5 positively charged R5X4-dualtropic viruses have switched to X4-tropism and therefore the X4-prediction resulted in a perfect fit. For the +4 positively charged NL-952.1 mutants RRQ and KKQ all algorithms gave different results and only PSSM_SINI_ was accurate enough to predict R5-monotropism for both mutants. The same issue was identified for the combination SRR/SKK and RTR/KTK. Here, none of the algorithms could predict the correct phenotype. The forecast of coreceptor usage was especially complicated for the R5-monotropic viruses with a +4 net charge in the context of virus lacking N-glycan g15. All viruses showed R5-monotropism in contrast to all four algorithms which voted for X4. Overall, the impact of R-to-K changes for coreceptor usage is not that significant as it is for viral infectivity per se. RRR-to-RRK for example leads to the addition of CCR5 usage but is not dependent on a specific R or K at any of the three positions 9, 10 or 11. On the other hand, V3 loop position 9 seems to be relevant for higher infectivity when occupied by R instead of K at positions 10 and 11. Thus R-to-K exchanges have a much higher impact on infectivity than on the selection of coreceptors.

## Conclusions

Predicting the HIV tropism correctly is nowadays an important part of antiviral treatment concepts [24]. Drugs like Maraviroc block highly specific the entry of R5 viruses [11]. To administer these drugs the coreceptor phenotype of circulating viruses should be known. Predictive algorithms are with no doubt a useful tool that will help to optimize patient-specific treatment procedures. The fact that arginine inside the V3 loop favored CXCR4 and lysine favored CCR5 usage might help to enhance chances for a more accurate coreceptor prediction. In addition to coreceptor usage we would suggest that the infectivity rate of viruses will be considered also in the analysis of HIV-1 sequences. Here amino acid R at V3 loop position 9 plays a more important role in contrast to amino acid K.

## Supporting Information

S1 Dataset Data of the ffu means and standard deviations for Fig. 2B, Fig. 3–6. (TXT)
